# Oncogenic microtubule hyperacetylation through BEX4-mediated sirtuin 2 inhibition

**DOI:** 10.1038/cddis.2016.240

**Published:** 2016-08-11

**Authors:** Jin-Kwan Lee, Janet Lee, Heounjeong Go, Chang Geun Lee, Suhyeon Kim, Hyun-Soo Kim, Hyeseong Cho, Kyeong Sook Choi, Geun-Hyoung Ha, Chang-Woo Lee

**Affiliations:** 1 Department of Health Sciences and Technology, SAIHST, Sungkyunkwan University, Seoul 06351, Korea; 2 Department of Molecular Cell Biology, Sungkyunkwan University School of Medicine, Suwon 16419, Korea; 3 Department of Pathology, University of Ulsan College of Medicine, Asan Medical Center, Seoul 05505, Korea; 4 Team of Radiation effect Research, Research Center, Dongnam Institute of Radiological and Medical Science, Busan 46033, Korea; 5 Department of Biochemistry and Molecular Biology, Ajou University School of Medicine, Suwon 16499, Korea

## Abstract

Five brain-expressed X-linked (BEX) gene members (BEX1–5) are arranged in tandem on chromosome X, and are highly conserved across diverse species. However, little is known about the function and role of BEX. This study represents a first attempt to demonstrate the molecular details of a novel oncogene BEX4. Among BEX proteins, BEX4 localizes to microtubules and spindle poles, and interacts with *α*-tubulin (*α*-TUB) and sirtuin 2 (SIRT2). The overexpression of BEX4 leads to the hyperacetylation of *α*-TUB by inhibiting SIRT2-mediated deacetylation. Furthermore, we found BEX4 expression conferred resistance to apoptotic cell death but led to acquisition of aneuploidy, and also increased the proliferating potential and growth of tumors. These results suggest that BEX4 overexpression causes an imbalance between TUB acetylation and deacetylation by SIRT2 inhibition and induces oncogenic aneuploidy transformation.

Microtubules are the largest filamentous components of the cytoskeleton, and have many different functions in eukaryotic cells. Recent advances point towards post-translational modifications of tubulin (TUB) as an effective mechanism for generating microtubule diversity, which depending on the cellular context varies with development, differentiation, cell compartment localization, and cell type.^1^ Among these modifications, acetylation of *α*-TUB, a well-known marker of stabilized microtubules, is important for microtubule interactions with microtubule-associated proteins (MAPs) and motor proteins, and is especially abundant on old microtubules with slow dynamics that are resistant to depolymerization.^2^ Interestingly, acetylation of *α*-TUB is frequently increased in many human cancers and has been proposed as a marker for sensitivity to microtubule-targeted anti-cancer drugs, such as paclitaxel.^3, 4^ Thus, acetylation of *α*-TUB should be strictly controlled by acetyltransferases and deacetylase during cell proliferation and growth. However, how microtubules remain highly acetylated during tumorigenesis and in cancer cells is currently unknown.

Microtubules are nucleated by duplicated centrosomes to generate two asters of highly dynamic microtubules to facilitate chromosome attachment and segregation. Chromosome segregation is mediated by a bipolar array of microtubules of varied lengths that continuously grow and shrink.^5^ There may be chromosome mis-segregation mostly caused by improper microtubules attachment to kinetochores, and it is a major source of aneuploidy.^6, 7^ Importantly, deregulation of *α*-TUB acetylation interferes with the normal timing of mitotic progression and mitotic checkpoint activation, leading to chromosome mis-segregation and instability.^8, 9^ As such, finely tuned regulation of TUB acetylation is crucial for microtubule dynamics in preventing chromosome instability and tumor progression.

Our initial attempt to find molecules involved in the regulation of mitotic checkpoint in response to spindle damage involved performing cDNA microarray analysis. Brain expressed X-linked gene 4 (BEX4) was found to be highly upregulated in aneuploid cells. *BEX* were initially found to be involved in the development of the nervous system and in neurological diseases.^10, 11^ Accumulating evidence, in addition, suggests the potential involvement of *BEX* in the development of cancer. For this family, BEX2 overexpression reportedly promoted the proliferation of cancer cells, whereas its downregulation promoted apoptotic cell death.^12, 13^ Microarray data demonstrated a higher expression level of BEX4 in primary tumors that had metastasized than in those that had not.^14^ In addition, mRNA levels of *BEX4* in human lung and liver tissues were reportedly higher than those in normal tissues.^15, 16^

Despite the above-mentioned evidence, the relationships between BEX and cancer are largely based on mRNA and protein expression data, and the mechanisms underlying gain- or loss-of-functional relevance are unknown. This study represents a first attempt to determine the molecular lesions caused by BEX4 expression.

## Results

### BEX4 expression allowed abnormal mitotic cells to adapt and become aneuploidy

**Figure 1:**
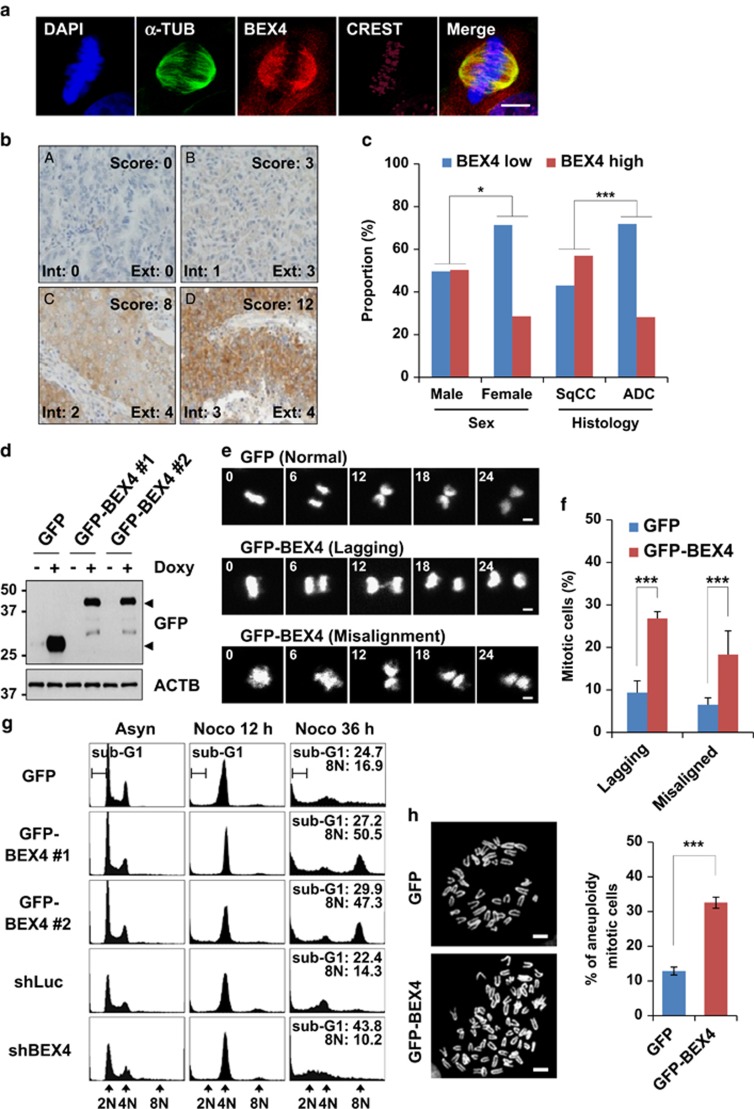
BEX4 expression led to abnormal mitosis and aneuploidy adaptation. (**a**) HeLa cells were fixed and co-stained with indicated antibodies. DAPI was used for staining DNA. Scale bars represent 10 *μ*m. (**b**) One-hundred ninety-one NSCLC patient samples (149 male, 42 female) were collected and subjected to IHC staining with antibodies against BEX4. A, NSCLC-1, score 0; B, NSCLC-2, score 3; C, NSCLC-3, score 8; D, NSCLC-4, score 12. Int, intensity scores; Ext, extent scores. (**c**) BEX4 expression in the 191 tissue samples was analyzed semiquantitatively by summing the products of staining intensity and tumor cell positivity scores. High expression was defined as a BEX4-IHC score of ⩾6, and low expression was defined as a BEX4-IHC score of ⩽5. Pearson's *χ*^2^ test was used to examine the statistical significance of some difference associated with BEX4. SqCC, squamous cell carcinoma; ADC, adenocarcinoma. *P = 0.012, ***P = 0.001. (**d**) Inducible cell lines expressing either control GFP or GFP-fused BEX4 were cultured in the absence (−) or presence (+) of doxycycline (2 *μ*g/ml), and 24 h post-treatment cells were lysed and immunoblotted with indicated antibodies. Arrowheads indicate GFP and GFP-BEX4. (**e**) Representative time-lapse microscopic images of HeLa cells expressing GFP or GFP-BEX4. Mitotic chromosomes were visualized using RFP signals resulting from H2B-RFP expression. Times are presented in minutes, and scale bars represent 10 *μ*m. (**f**) Quantitative comparison of GFP- and GFP-BEX4-expressing HeLa cells showing lagging chromosome or misaligned chromosome. Results are presented as the means±S.E.Ms of three independent experiments. ***P<0.001. (**g**) HeLa cells transfected with shBEX4 plasmid, and doxycycline treated GFP or GFP-BEX4 inducible cells were treated with nocodazole (100 ng/ml). At the times indicated, cells were harvested, and stained with propidium iodide. DNA contents were analyzed by flow cytometry. (**h**) Primary MEFs were infected with recombinant retroviruses encoding GFP or GFP-BEX4 (two independent experiments were performed). At 48 h post-infection, cells were treated with colcemid for 6 h. Chromosomes were spread and visualized by DAPI staining. Scale bars represent 5 μm. Chromosome spread percentages were calculated using at least 100 cells per experiment. ***P<0.001

To gain an insight into the functional relevance of BEX4 expression, we monitored the subcellular distribution of BEX4 using the affinity purified rabbit polyclonal antibody against a peptide from human BEX4 (EIKRKTREQQMRHYMRFQ; Supplementary Figure S1). Immunofluorescence analyses revealed localization of the BEX4 at microtubules and spindle poles (Figure 1a) and also at nucleus and cytoplasm (Supplementary Figure S2a). Depletion of BEX4 expression by shBEX4 transfection reduced the BEX4 levels at microtubules and spindle poles (Supplementary Figure S2b).

To examine the relevance of BEX4 gain-of-function in human cancers, we compared BEX4 levels in 191 patients with non-small cell lung cancer (NSCLC), including 107 squamous cell carcinoma (SqCC, 56.0%), 71 adenocarcinoma (ADC, 37.2%), 9 large cell carcinoma (4.7%), 2 adenosquamous cell carcinoma (1.0%), and 2 sarcomatoid carcinoma (1.0%), immunohistochemically using anti-BEX4 antibody. The clinicopathologic features of NSCLC displayed clear cytoplasmic and weak nuclear BEX4 expression (Figure 1b). In fact, BEX4 was highly expressed in 45.5% of the tumor specimens and 54.5% of tumor specimens displayed no or low BEX4 expression (high and low expression were defined as IHC score of ⩾6 and ⩽ 5, respectively). Interestingly, the high expression rate was greater in male patients (50.3%) than in female patients (28.6%). Furthermore, the high expression rate was significantly greater for SqCC samples than those for ADC (57% *versus* 28.2% *P*=0.001) (Figure 1c). In contrast, immunohistochemical analysis for BEX4 expression in normal lung tissues mostly had a cytoplasmic distribution and a low expression rate (<3) in pneumocytes and respiratory epithelial cells (Supplementary Figure S3a and b).

To examine the growth property changes induced by BEX4 overexpression, we generated doxycycline-inducible HeLa cells expressing control GFP or GFP-fused BEX4 (Figure 1d), and compared the overall cell cycle progressions using time-lapse microscopy. Interestingly, we found that BEX4 overexpression led to high lagging and misaligned chromosome rates (Figures 1e and f), indicating that the deregulation of BEX4 expression contributes to mitotic abnormality.

To determine if BEX4 expression affects the mitotic checkpoint and chromosome segregation, HeLa cells over- or under-expressing BEX4 were cultured in the absence or presence of nocodazole and subjected to fluorescence activated cell sorting (FACS) analysis. Most of the control GFP or shLuc cells arrested at mitosis by nocodazole eventually died (Figure 1g, sub-G1 population), whereas remaining cells exited arrest in a process called ‘mitotic adaptation' or ‘mitotic slippage' and became aneuploid (Figure 1g, 8N population).^17, 18, 19^ However, extended incubation of BEX4-overexpressing cells in the presence of nocodazole led to the accumulation of aneuploid cells and relatively few apoptotic cells. BEX4-depleted cells were also arrested in mitosis, but more cells died instead of becoming aneuploid (8N). Far fewer BEX4-depleted cells than controls survived as an aneuploid population. These observations suggest chromosomal aneuploidy in response to spindle damage seems to be directly regulated by BEX4 levels. We also examined the effect of BEX4 expression in primary mouse embryonic fibroblasts (MEFs) infected with recombinant retrovirus expressing GFP or GFP-BEX4 (Figure 1h). Metaphase chromosome spreading analysis revealed that aneuploid populations were significantly higher in BEX4-overexpressing MEFs than in control MEFs, showing that BEX4 expression induces acute and severe chromosomal aneuploidy.

### BEX4 regulates acetylation of *α*-TUB

**Figure 2:**
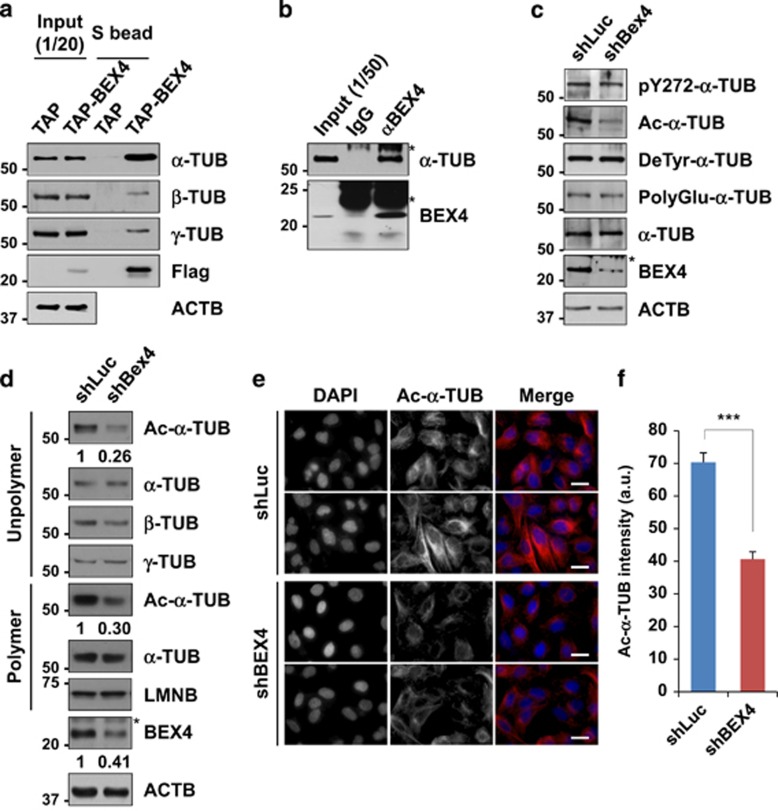
BEX4 interacts with microtubules and maintains acetylation of *α*-TUB. (**a**) HeLa cells were transfected with either control TAP- or TAP-BEX4-expressing plasmids. Lysates were incubated with streptavidin sepharose beads (S beads). Bound proteins were resolved by SDS-PAGE and immunoblotted. (**b**) HeLa cell lysates were immunoprecipitated with anti-BEX4 antibody and immunoblotted. Asterisk denotes immunoglobulins. (**c**) Lysates from U2OS cells expressing either shLuc or shBEX4 were immunoblotted. Asterisk denotes a nonspecific band. Ac, acetylated; pY272, phospho-Y272; PolyGlu, polyglutamylated; DeTyr, detyrosinated. (**d**) U2OS cells were transfected with shLuc or shBEX4. After 24 h, cellular extracts were separated into unpolymerized (unpolymer) and polymerized TUB (polymer) fractions, and then immunoblotted. The numbers at the bottom of blots indicate the relative intensity of bands normalized *versus* actin expression. The asterisk represents a nonspecific band. Ac, acetylated. (**e**) U2OS cells expressing shLuc or shBEX4 were fixed, and co-stained with anti-acetylated-*α*-TUB (Ac-*α*-TUB) and DAPI. Scale bars represent 20 *μ*m. (**f**) Quantification of the relative mean fluorescence intensities (a.u., arbitrary units) of acetylated-*α*-TUB (Ac-*α*-TUB) in shLuc (*n*=101) or shBEX4 (*n*=126) transfected cells as determined using Image J software. Results are presented as the mean±S.E.M. of three independent experiments. ***P<0.001

Deregulation of TUB acetylation leads to chromosome mis-segregation and aneuploidy. In addition, acetylation of *α*-TUB is frequently elevated in many human cancers,^20^ and hyperacetylation of *α*-TUB interrupts the normal timing of mitotic progression and confers resistance to microtubule inhibitors.^9, 21^ To test the possibility that BEX4 formed complexes with microtubule subunits and regulated their modification, lysates of HeLa cells stably expressing tandem affinity purification (TAP)-tagged BEX4 or TAP alone were incubated with the streptavidin beads and analyzed by immunoblotting. Interestingly, BEX4 formed a complex with *α*-TUB and very weakly with *β*-TUB and *γ*-TUB (Figure 2a). Immunoprecipitation assay revealed a complex comprised of BEX4 and endogenous *α*-TUB (Figure 2b).

Microtubule dynamics are regulated by post-translational modifications of TUB subunits and these include phosphorylation, acetylation, polyglycosylation, polyglutamylation, detyrosination/tyrosination, and polyamination.^22^ To investigate the mechanism responsible for the interaction between BEX4 and *α*-TUB, we determined the influence of BEX4 expression on the expression levels and post-translational modifications of *α*-TUB (Figure 2c). The acetylation of *α*-TUB, particularly at lysine 40, is crucial for microtubule polymerization, with extent of microtubule acetylation being related to microtubule stability.^23, 24, 25, 26^ The phosphorylation of *α*-TUB tyrosine 272 generally occurs at the final stages of functional differentiation, which assists in permanently stabilizing microtubules.^27^ The tyrosination state of *α*-TUB regulates the activity of depolymerizing mitotic centromere-associated kinesin, which is essential for proper chromosome segregation in anaphase.^28, 29^ In turn, polyglutamylation of *α*-TUB induces enzymatic microtubule severing and might be critical for control of the length of the mitotic spindle.^30, 31^ Interestingly, RNA interference-mediated depletion of BEX4 had no significant effect on the levels of phosphorylated, tyrosinated or polyglutamylated *α*-TUB, but resulted in a clear decrease in the level of *α*-TUB acetylation (Figure 2c).

To examine the effect of BEX4 depletion on microtubule dynamics, we monitored the levels of acetylated *α*-TUB levels in polymerized and unpolymerized TUB fractions of BEX4-depleted cells (Figure 2d). BEX4-depleted cells displayed less acetylated *α*-TUB in the monomer and polymer subunit fractions than in control cells. The levels of total *α*-, *β*-, and *γ*-TUB were unaffected by BEX4 depletion. Immunofluorescence analysis showed that BEX4 depletion significantly decreased the fluorescence intensity of acetylated *α*-TUB compared with the control cells (Figures 2e and f). These results suggest that BEX4 has an important role in regulating microtubule acetylation.

### BEX4 regulates microtubule acetylation through inhibition of the *α*-TUB deacetylase sirtuin 2 (SIRT2)

**Figure 3:**
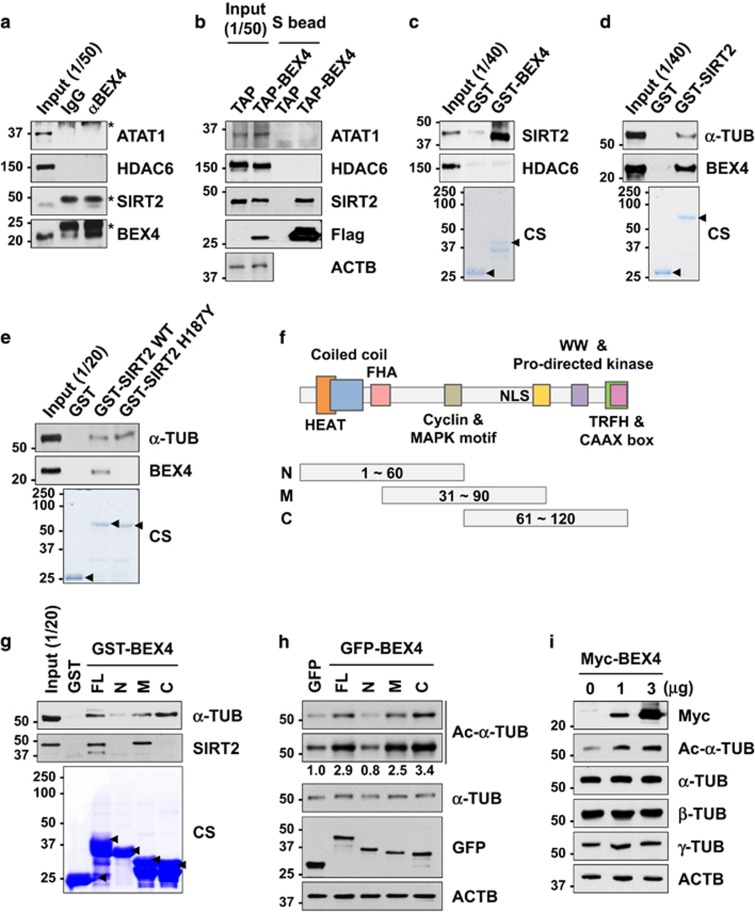
BEX4 interacts with SIRT2 and leads to the hyperacetylation of *α*-TUB. (**a**) U2OS cell lysates were immunoprecipitated with anti-BEX4 antibody and immunoblotted with indicated antibodies. Asterisk denotes immunoglobulins. (**b**) U2OS cells were transfected with either control TAP- or TAP-BEX4-expressing plasmids, and lysates were incubated with streptavidin beads (S bead). Bound proteins were resolved by SDS-PAGE and immunoblotted. (**c**) U2OS cell extracts were incubated with beads bound to the GST alone or GST-BEX4. Bound proteins were resolved and immunoblotted. The arrows denote purified GST and GST-BEX4 proteins, respectively. (**d**) Purified GST, and GST-SIRT2 proteins were incubated with U2OS cell extracts, and pull-downed with beads. Bound proteins were resolved and immunoblotted. The arrows denote purified GST and GST-SIRT2 proteins, respectively. (**e**) Pull-down assay using U2OS lysate and either GST, GST-SIRT2 wild type (WT), or GST-SIRT2 H187Y mutant. Immunoblot was performed with indicated antibodies. (**f**) Schematic amino-acid sequence signatures of BEX4. (**g**) U2OS cell extracts were incubated with beads bound to the GST, GST-BEX4 (full-length; FL), GST-BEX4 N, GST-BEX4 M, or GST-BEX4 C. Bound proteins were resolved and immunoblotted. The arrows denote purified GST and GST, GST-BEX4 (FL), and GST-BEX4 mutant proteins (N, M, and C), respectively. (**h**) U2OS cells were transfected with GFP and GFP-BEX4 (FL, N, M, and C) expressing plasmid. Each sample was immunoblotted with indicated antibodies. The numbers at the bottom of blots indicate the relative intensity of bands normalized *versus* α-TUB expression. Ac, acetylated. (**i**) U2OS cells were transfected with 0, 1, and 3 *μ*g of Myc-tagged BEX4-expressing plasmid. Each sample was immunoblotted with indicated antibodies. CS, coomassie brilliant blue staining. Ac, acetylated

The acetylation of polymerized *α*-TUB at lysine 40 is tightly controlled by the enzymatic activity of acetyltransferases like ATAT1, and negatively regulated through the activity of deacetylases HDAC6 and SIRT2.^32, 33, 34^ We hypothesized that BEX4 may promote microtubule hyperacetylation by either enhancing the activation of acetyltransferase or inhibiting TUB deacetylase. A possible interaction between BEX4 and ATAT1, or HDAC6 and SIRT2 was initially examined by immunoprecipitation and TAP pull-down assay (Figures 3a and b). These assays show that BEX4 formed a complex only with SIRT2.

Next, we generated glutathione S-transferase (GST)-tagged fusion proteins (GST-BEX4 and GST-SIRT2) and incubated them with U2OS cell extracts (Figures 3c and d). Immunoprecipitation and pull-down assays revealed that the interaction of BEX4 with SIRT2 but not with HDAC6 or ATAT1. A subsequent pull-down assay showed that SIRT2 wild-type, but not the H187Y deacetylase activity dead mutant, formed a complex with BEX4, whereas both SIRT2 wild-type and H187Y mutants interact with *α*-TUB (Figure 3e), indicating that BEX4 may compete with SIRT2 for *α*-TUB binding.

To define the domains responsible for the BEX4-TUB or BEX4-SIRT2 interaction, we generated variant GST-BEX4 fusion proteins each comprised of three different regions in the molecule: GST-BEX4 N-terminal (N; 1~60 a.a), middle (M; 31~90 a.a), and C-terminal (C; 61 ~120 a.a). BEX4 is highly conserved among various species, and has several consensus amino-acid sequence motifs. These include the HEAT domain ligand, coiled-coil domain, fork-head-associated (FHA) phosphopeptide ligand, cyclin-binding motif, mitogen-activated protein kinase (MAPK)-binding motif, WW ligand class IV, proline-directed kinase-binding motif, and CAAX box (Figure 3f). Pull-down assays revealed that *α*-TUB bound to GST-BEX4 C and very weakly to GST-BEX4 M. Interestingly, GST-BEX4 M only formed a complex with SIRT2 (Figure 3g), and overexpression of GFP-BEX4 M and C increased acetylation of *α*-TUB (Figure 3h). In addition, the overexpression of increasing amounts of BEX4 concomitantly augmented the levels of acetylated *α*-TUB, whereas the overall levels of *α*, *β*, and *γ*-TUB were unaffected (Figure 3i). Collectively, our results indicate that BEX4 may compete with SIRT2 for *α*-TUB binding and regulate the status of acetylation of *α*-TUB.

### Overexpression of BEX4 leads to *α*-TUB hyperacetylation via inhibition of SIRT2 activity

**Figure 4:**
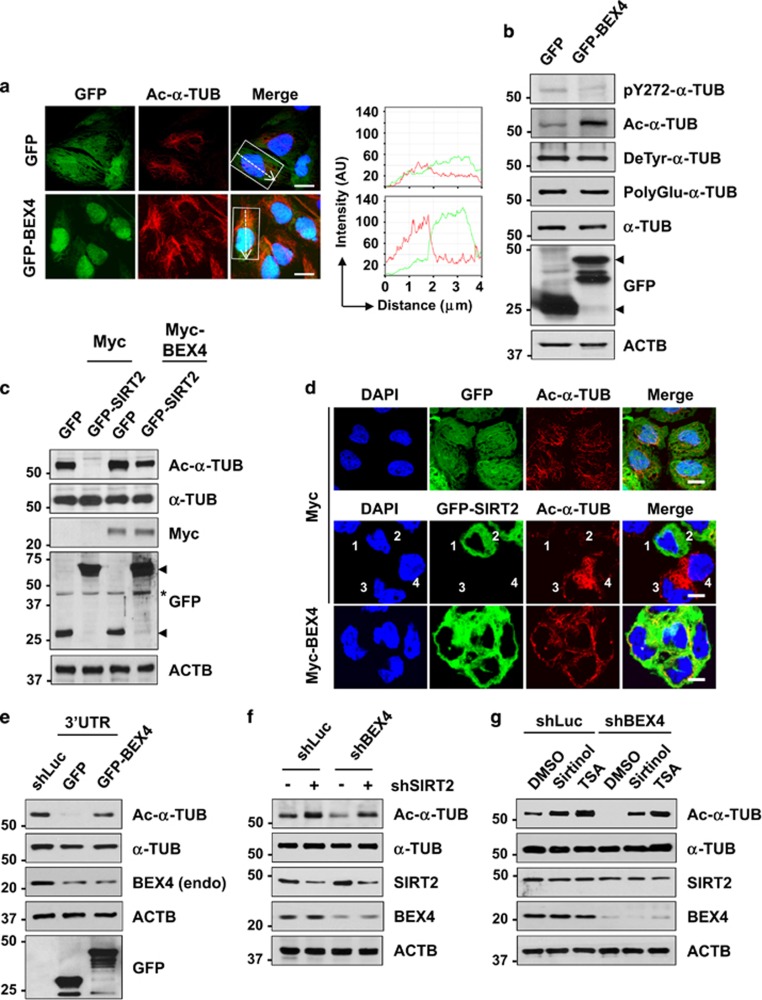
Overexpression of BEX4 induces hyperacetylation of *α*-TUB through SIRT2 inhibition. (**a**) U2OS cells transfected with GFP or GFP-BEX4 were fixed with 4% paraformaldehyde, and stained with anti-acetylated-*α*-TUB (Ac-*α*-TUB) antibody and DAPI to visualize DNA. Right panels represented the intensity of fluorescence signal from insets in left panel. Scale bars represent 10 *μ*m. (**b**) Lysates from U2OS cells expressing control GFP or GFP-BEX4 were immunoblotted with indicated antibodies. Ac, acetylated; pY272, phospho-Y272; PolyGlu, polyglutamylated; DeTyr, detyrosinated. (**c**) Lysates from U2OS cells transfected with a control Myc or Myc-tagged BEX4 expression plasmid individually or in combination with GFP or GFP-fused SIRT2 expression plasmid were analyzed by immunoblotting using indicated antibodies. Ac, acetylated. (**d**) U2OS cells from Figure 3c were fixed with 4% paraformaldehyde, and stained with anti-acetylated-*α*-TUB (Ac-*α*-TUB) antibody and DAPI to visualize DNA. GFP-tagged SIRT2-derived fluorescence is green. Scale bars represent 10 *μ*m. (**e**) U2OS cells were transfected with 3′ untranslated region BEX4 shRNA (3′UTR; targeting BEX4 3′UTR) in combination with shLuc, GFP- or GFP-BEX4-expressing plasmid, and immunoblotted with indicated antibodies. Ac, acetylated. (**f**) U2OS cells transfected with control shLuc or shBEX4 in combination with shSIRT2 were immunoblotted. Ac, acetylated. (**g**) U2OS cells transfected with control shLuc or shBEX4 with or without sirtinol or TSA were immunoblotted with indicated antibodies. Ac, acetylated

To further assess the effect of BEX4 expression on *α*-TUB modification, U2OS cells were transfected with expression plasmids for control GFP or GFP-BEX4. Analysis of subcellular localization of BEX4 revealed that BEX4 overexpression caused a significant increase in acetylated *α*-TUB (Figure 4a). In addition, BEX4 overexpression augmented the level of acetylated *α*-TUB, but not the levels of phosphorylated, tyrosinated, or polyglutamylated *α*-TUB (Figure 4b).

To examine whether the hyperacetylation of *α*-TUB by BEX4 overexpression was due to its competition with SIRT2, U2OS cells were transfected with a control Myc or Myc-tagged BEX4 expression plasmid individually or in combination with GFP or GFP-fused SIRT2 expression plasmid. The overexpression of SIRT2 sharply reduced the level of acetylated *α*-TUB as previously reported,^33^ whereas the overexpression of BEX4 in the SIRT2-overexpressing cells significantly recovered the levels of acetylated *α*-TUB (Figure 4c). Further immunofluorescence analysis revealed that the overexpression of GFP-SIRT2 in control Myc expression vector transfected cells (middle panels, numbers 1 and 2) significantly reduced the level of acetylated *α*-TUB compared with those of GFP (upper panels) and the GFP-SIRT2-untransfected cells (middle panels, numbers 3 and 4) (Figure 4d). However, cells overexpressing both GFP-SIRT2 and Myc-BEX4 (lower panels) showed the clear recovery of acetylated *α*-TUB levels.

To verify the effect of BEX4-SIRT2 interaction on *α*-TUB acetylation, U2OS cells were transfected with control Luciferase (shLuc) or BEX4 shRNA, which targeted the 3′ untranslated region of BEX4 (3′UTR), in combination with GFP or GFP-BEX4. In condition that depletion of BEX4 reduced the level of acetylated *α*-TUB, the reintroduction of GFP-BEX4 recovered the induction of acetylated *α*-TUB (Figure 4e). We next co-transfected U2OS cells with control shLuc or shBEX4 with or without shSIRT2 and the cells showed a pattern similar to that of BEX4 overexpression (Figure 4f). To determine whether hypoacetylation of *α*-TUB by BEX4 depletion was due to elevated activity of SIRT2, we exposed the cells transfected with shLuc and shBEX4 to sirtinol (a SIRT2 inhibitor) and trichostatin A (TSA; an HDAC inhibitor). Acetylation levels of *α*-TUB were recovered by treatment with sirtinol and TSA in BEX4 depleted cells. To determine whether SIRT2 inhibition could show similar results from BEX4 overexpression, SIRT2 inhibitors, sirtinol and AGK2, were treated in combination with nocodazole. Prolonged exposure to SIRT2 inhibitors with nocodazole induced aneuploidy as expected (Supplementary Figure S4). Taken together, these results suggest that the BEX4 had a considerable influence on the regulation of *α*-TUB acetylation by potentially controlling SIRT2 activity.

### BEX4 overexpression increases tumor proliferation potential and growth

**Figure 5:**
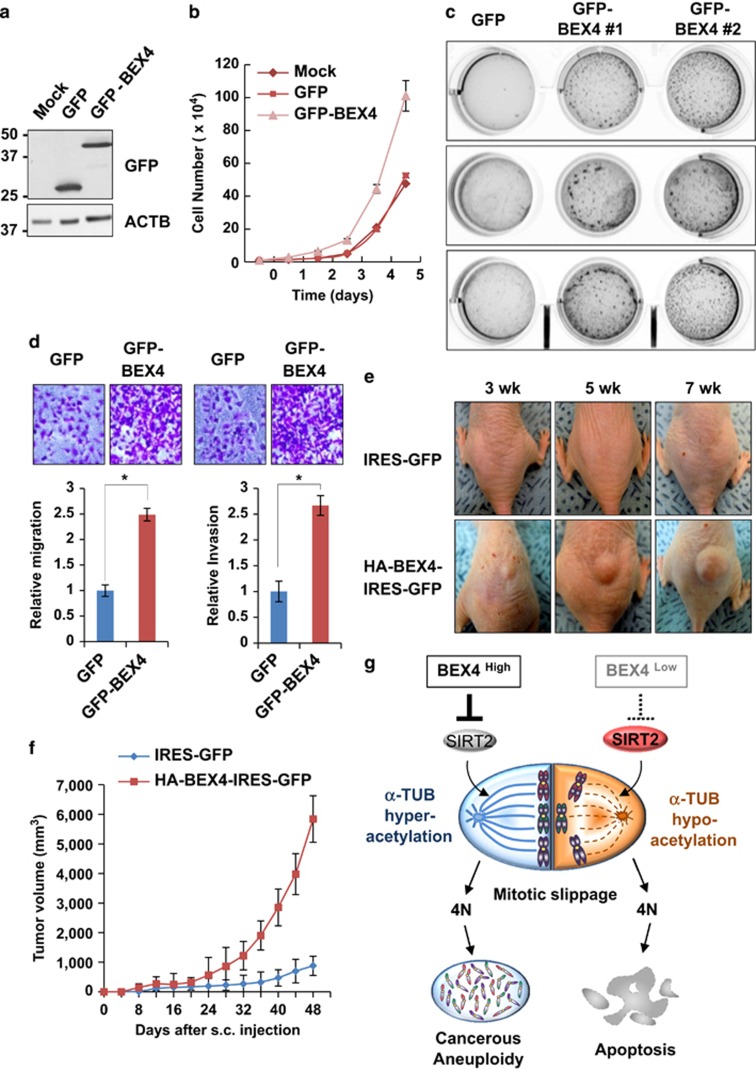
BEX4 increases the proliferating potential and contributes to the development of tumors. (**a**) HeLa cells were transfected with GFP- or GFP-BEX4-expressing plasmid, and the level of GFP and GFP-fused BEX4 proteins were determined by immunoblotting. (**b**) Equal numbers (1 × 10^4^ cells) of mock, GFP, and GFP-BEX4 transfected cells were seeded in six-well plates. Each cell numbers were counted for five consecutive days. Error bars indicate S.D. from three independent experiments. (**c**) Inducible cell lines expressing either control GFP or GFP-fused BEX4 were cultured on soft agar following 24 h post-treatment with doxycycline (2 *μ*g/ml). Four weeks after culture, the soft agar plates were stained with crystal violet. (**d**) GFP- or GFP-BEX4-expressing HeLa cells were subjected to transwell migration (left panels) and matrigel invasion assays (right panels). Data shown are mean±S.D. of three independent experiments. *P<0.05. (**e**) 1 × 10^6^ BALB/3T3 (immortalized mouse fibroblast cell line) cells infected with retrovirus expressing a control GFP or GFP plus HA-tagged BEX4 were inoculated subcutaneously into the backs of athymic nude mice (eight mice per group). Animals were then monitored and photographed every 2 weeks. (**f**) Tumor sizes were measured in three dimensions using an electronic caliper at 2-day intervals. (**g**) A model of BEX4-mediated *α*-TUB hyperacetylation by SIRT2 inhibition. Refer to the Discussion for details

To examine the effect of BEX4 expression on cell proliferation and growth, HeLa cells were transfected with expression plasmids encoding GFP-BEX4 or GFP (Figures 5a and b). Cells overexpressing GFP-BEX4 proliferated much faster than control GFP-overexpressing or non-transfected cells. We next tested whether BEX4 expression altered the ability of cells to grow anchorage independently, which is a hallmark of cancer cells. Soft agar assays using doxycycline-inducible GFP- and GFP-BEX4-expressing HeLa cells revealed that cells expressing GFP-BEX4 formed large number of colonies when seeded at low density in agar medium in presence of doxycycline, whereas control GFP cells formed far fewer colonies (Figure 5c). In addition, we tested whether overexpression of BEX4 affected cell migration and invasion capability. Transwell migration and invasion assays using HeLa cells expressing GFP or GFP-BEX4 revealed that cells overexpressing GFP-BEX4 were markedly less able to migrate and invade compared with those expressing GFP (Figure 5d).

Next, to further examine the effect of BEX4 overexpression *in vivo*, BALB/3T3 cells (an immortalized non-tumorigenic cell line) were infected with retroviruses expressing control GFP or GFP-fused BEX4 (Supplementary Figure S5). Athymic nude mice subcutaneously injected with BALB/3T3 cells-expressing GFP-BEX4 showed significant higher growth under the skin of 20-fold than cells expressing GFP (Figures 5e and f). These results suggest that BEX4 overexpression contributes to anchorage independent growth and tumorigenesis. The collective results indicate that BEX4 expression provides a novel oncogenic signal by enabling the acquisition of chromosomal aneuploidy and inducing the hyperacetylation of *α*-TUB through SIRT2 inhibition.

## Discussion

TUB acetylation modulates the ability of microtubules to bind to MAPs and motor proteins, and may regulate microtubule stability and function.^35, 36^ During metaphase, acetylated TUB is enriched at interpolar and kinetochore microtubules, and becomes concentrated on the midbody during telophase and cytokinesis.^1, 35, 37^ These observations support the view that finely-tuned regulation of TUB acetylation is crucial for bipolar attachment of the chromosome to the kinetochore and for cytokinesis. Thus, deregulation of TUB acetylation leads to chromosome mis-segregation and aneuploidy, and studies have shown that acetylation of *α*-TUB is frequently elevated in many human cancers.^20^ This study was undertaken to elucidate the role of BEX4 in the acquisition of aneuploidy. The results reveal a molecular basis of *α*-TUB hyperacetylation during tumorigenesis. BEX4 promotes the hyeracetylation of *α*-TUB by inhibiting SIRT2-mediated deacetylation (Figure 5g). Importantly, BEX4 expression led to significant aneuploidy and increase tumor growth. This raises the significant possibility that the overexpression of BEX4 contributes to the development of oncogenic TUB hyperacetylation through cross talk with *α*-TUB and SIRT2.

Microtubule inhibitors, stabilizing (paclitaxel) and destabilizing (vincristine and vinblastine) agents are widely used drugs for diverse cancer treatments. These microtubule inhibitors bind to TUB, prevent cells undergoing normal mitosis, and subsequently induce apoptotic cell death. Hyperacetylation of *α*-TUB by SIRT2 downregulation confers resistance to microtubule inhibitors by prolonged mitotic arrest, and leads to chromosome instability and aneuploidy, rather than apoptotic cell death.^9, 21^ In this study, BEX4 protein mainly localized to a number of mitotic structures including microtubules, contractile rings, and midbodies. Furthermore, BEX4 expression seemed to critically determine whether cells underwent apoptosis or adapted to aneuploidy induced by a microtubule inhibitor.

Both SIRT2 and HDAC6 deacetylate TUB and colocalize with the microtubule network.^9^ Depletion of either SIRT2 or HDAC6 resulted in an increase in acetylated *α*-TUB. However, TUB hypoacetylation or deacetylation does not directly lead to formation of stable microtubules stability; increased TUB acetylation by TSA, an inhibitor of HDAC6, does not promote microtubule stabilization.^38^ Thus, it is still unclear how hypoacetylation and/or deacetylation of *α*-TUB by SIRT2 and HDAC6 are involved in any TUB-related events. Recently, it was reported that downregulation of SIRT2 confers resistance to microtubule inhibitors by abnormally prolonging mitotic arrest and thus compromising the cell death pathway after mitotic slippage.^39^ Indeed, in the present study, abnormal mitotic cells, from treatment with either a microtubule inhibitor or having a mitotic checkpoint defect, were able to adapt and become aneuploidy when BEX4 was overexpressed. In addition, SIRT2 deacetylates BUB1B during prometaphase and inhibits its anaphase-promoting complex/cyclosome (APC/C)-dependent proteolysis.^39^ These events regulate timing in anaphase entry. Interestingly, the depletion of BUB1B abnormally prolonged spindle assembly checkpoint (SAC) activation, which is also observed in SIRT2-depleted cell.^9, 39^ Prolonged mitotic arrest at SAC is a well-known requirement for mitotic catastrophe and in conferring sensitivity to microtubule inhibitors. Therefore, BEX4 expression and SIRT2 activity for *α*-TUB hypoacetylation or deacetylation may profoundly influence tumorigenesis and the acquisition of preneoplastic aneuploidy.

Acetylated microtubules are far more stable and resistant to drug-induced depolymerization than non-acetylated microtubules.^4^ Furthermore, one of the mechanisms of acquired resistance to microtubule inhibitors involves the expression-mediated upregulation of TUB isotypes, which inhibit the actions of these agents.^3^ For this reason, microtubule inhibitors fail to affect microtubule dynamics effectively when microtubule destabilizing agents are employed in an anti-cancer strategy. Presently, inhibition of BEX4 expression reduced acetylated *α*-TUB levels and deregulated microtubule dynamics. Therefore, inhibition of BEX4 expression in combination with microtubule destabilizing agent treatment could improve treatment efficacy in terms of inhibiting the proliferation of tumor cells. Accordingly, these results suggest that the regulation of BEX4 expression provides an attractive anti-cancer strategy.

BEX4 overexpression provided a proliferative advantage to abnormal mitotic cells and protected them against spindle damage-induced apoptosis. Earlier studies have suggested that inappropriate signaling to stabilize and organize centrosomes and their microtubules, respectively, leads to aberrant cell cycle regulation, overriding the mitotic checkpoint, and chromosome aneuploidy.^7^ These suggestions indicate the finely tuned regulation of microtubule dynamics may protect a cell from excess microtubule damage that might occur during sustained mitotic arrest and compromise cell survival if the cell fails to return mitotic cell cycle progression. Interestingly, BEX4 expression reduced the population of nocodazole-induced apoptotic cells and increased the accumulation of aneuploid cells, which are prone to oncogenic transformation. Thus, to prevent increased propensity for oncogenic transformation in a normal cell, BEX4 expression must be stringently regulated by an as-yet-unidentified mechanism. However, the constitutive expression of BEX4 resulted in the severe dysregulation of microtubule dynamics, which are known to be associated with cancer. Taken together, our results indicate that increased BEX4 expression increases oncogenic potential as it acts to deregulate microtubule acetylation and chromosomal integrity.

## Materials and methods

### Antibodies

Rabbit polyclonal antibodies against C-terminal polypeptides 89-106 of BEX4 were commercially generated (Young In Frontier, Seoul, Rep. of Korea). The other antibodies used in this study were anti-acetylated-*α*-TUB (Ac-*α*-TUB) (6-11B-1), anti-ATAT1 (B-20), anti-*α*-TUB (B-7), anti-*β*-TUB (H-235), anti-*γ*-TUB (H-183), anti-green fluorescence protein (GFP; B-2), anti-HDAC6 (H-300), anti-SIRT2 (H-95) (Santa Cruz Biotechnology, Shanghai, China), anti-ACTB (AC-15), anti-Flag (M2) (Sigma-Aldrich, Seoul, Korea), anti-LMNB (ab16048), anti-phospho-Y272 (pY272) *α*-TUB (EP13342Y) (Abcam, Cambridge, UK), anti-polyglutamylated (PolyGlu) *α*-TUB (GT335) (Adipogen, Liestal, Switzerland), anti-detyrosinated (deTyr) *α*-TUB (AB3201) (Merck Millipore, Daejeon, Rep. of Korea), anti-HA (12CA5) (Roche, Seoul, Rep. of Korea), anti-Myc (A190) (Bethyl, Montgomery, TX, USA), and Alexa Fluor (Invitrogen, Carlsbad, CA, USA).

### Patients

One-hundred ninty-one Korean patients with NSCLC that underwent pulmonary resection for primary tumor at Seoul National University Hospital between January 1995 and January 2000 and with available formalin-fixed paraffin-embedded (FFPE) tissue were enrolled. Representative 2 mm-diameter cores were taken from FFPE tissue blocks to construct tissue microarrays. The Institutional Review Board of Seoul National University Hospital approved the study.

### Evaluation of BEX4 immunohistochemistry (IHC)

The cytoplasmic staining of BEX4 antibody was scored using a semiquantitative scoring system that took into account staining intensities and extents in 191 NSCLC specimens. Staining intensity was scored as no staining=0, weak staining=1, moderate staining=2, and strong staining=3, and the extent of staining as 0%=0, 1–10%=1%, 11–50%=2, 51–80%=3, and 81–100%=4. BEX4-IHC results were scored by multiplying the product of intensity by extent scores, which resulted in a minimum score of 0 and a maximum score of 12. Pearson's *χ*^2^ test was used to determine the statistical significances of differences.

### Cell line culture and establishment of inducible cell lines

MEFs were isolated from 13.5-dpc (day post coitum) embryos of wild-type mice. MEFs were stocked and cultured for maximum of five passages. HeLa (CCL-2) and U2OS (HTB-96) cell lines were purchased from ATCC (American Type Culture Collection, Manassas, VA, USA). HeLa tet-on cell line was purchased from Clontech (Seoul, Rep. of Korea). HeLa, HeLa tet-on and MEFs were cultured in Dulbecco's modified Eagle's medium (Dulbecco's modified Eagle's medium (DMEM); WelGENE, Gyeongsan, Rep. of Korea) containing 10% fetal bovine serum (FBS; Hyclone, Logan, UT, USA). U2OS cells were cultured in RPMI 1640 (WelGENE) containing 10% FBS. To generate HeLa cells inducibly expressing GFP or GFP-BEX4 proteins, HeLa tet-on cells were transfected with pTRE2hyg-GFP or pTRE2hyg-GFP-BEX4. Colonies showing resistance to hygromycin (20 *μ*g/ml) were clonally isolated. GFP and GFP-BEX4 proteins were induced by treating cells with doxycycline (2 *μ*g/ml) for 24 h.

### FACS analysis

Cells were harvested at indicated times post-treatment and then fixed and stained with propidium iodide. The DNA contents of 10 000 cells per sample were analyzed using a FACS Canto II cytometer (Becton Dickinson, Seoul, Rep. of Korea).

### Affinity purification of TAP-tagged protein complexes

The TAP purification method was previously described.^40^ The TAP tag consists of S-tag, Flag epitope, and streptavidin-binding peptide.

### Retrovirus production

Retroviral vectors were transfected into 293 T cells in combination with pCL-ECO. Two days after transfection, retroviral supernatants were collected and filtered through a 0.45-*μ*m filter.

### Pull-down and *in vitro* binding assays

These methods were previously described.^41^

### *In vivo* tumorigenesis assay

This study was reviewed and approved by the Institutional Animal Care and Use Committee (IACUC) of Sungkyunkwan University School of Medicine (SUSM). SUSM is an Association for Assessment and Accreditation of Laboratory Animal Care International (AAALAC International) accredited facility and abide by the Institute of Laboratory Animal Resources (ILAR) guide. Six-week-old male athymic BALB/c nude mice were purchased from Charles River Laboratories (Seoul, Rep. of Korea). BALB/3T3 cells transduced with retrovirus were resuspended in PBS at 1 × 10^7^ cells/ml and then injected subcutaneously into mice. Tumor volumes (mm^3^) were measured using an electronic caliper and were calculated using length × width^2^ × 0.5.

### Metaphase chromosome spreading assays

MEFs were treated with colcemid (100 ng/ml; Gibco, Carlsbad, CA, USA) for 6 h and mitotic cells were collected by shake-off. These cells were then incubated in a hypotonic buffer and fixed with Carnoy's solution. Cells in Carnoy's solution were dropped onto glass slides and dried at room temperature. Slides were stained with 4',6-diamidino-2-phenylindole (DAPI), mounted, and analyzed by fluorescence microscopy.

### Cell proliferation and soft agar assays

Cells were harvested by trypsinization, and viability assessed by trypan blue exclusion under a phase-contrast microscope. Equal number of cells were seeded. Cell numbers were determined by hemocytometric counting for 5 consecutive days. For the soft agar assay, cells were treated with doxycycline (2 *μ*g/ml) for 24 h, and mixed with 0.33% agarose in DMEM and overlaid on 0.5% agarose in a 12-well plate. After 4 weeks, colony formation was examined by staining colonies with 0.005% crystal violet (Sigma-aldrich) and stained colonies were counted using an inverted fluorescent microscope (Nikon, Seoul, Rep. of Korea).

### Transwell migration and invasion assays

Migration assays were performed using uncoated cell culture inserts with 8-μm pores (Greiner Bio-One, Kremsmünster, Austria). Invasion assays were carried out using cell invasion assay kits (Merck Millipore). Both assays were performed according to the manufacturer's instructions. Each result was evaluated by staining the cells using 0.05% crystal violet.

### Statistical analysis

All experimental data are reported as means±S.D. or S.E.M. by Excel software (Microsoft, Redmond, WA, USA). Student's *t*-test was used for statistical comparisons.

## Abbreviations

BEX: brain-expressed X-linked
TUB: tubulin
SIRT2: sirtuin 2
ATAT1: alpha tubulin acetyltransferase 1
HDAC6: histone deacetylase 6
GST: glutathione S-transferase
MAP: microtubule-associated protein
NSCLC: non-small cell lung cancer
SqCC: squamous cell carcinoma
ADC: adenocarcinoma
IHC: immunohistochemistry
Ac: acetylated
PolyGlu: polyglutamylated
DeTyr: detyrosinated
CS: coomassie brilliant blue staining
FACS: fluorescence activated cell sorting
MEF: mouse embryonic fibroblast
TAP: tandem affinity purification
FHA: fork-head associated
MAPK: mitogen-activated protein kinase
3'UTR: 3′ untranslated region
TSA: trichostatin A
DMEM: Dulbecco's modified Eagle's medium
FBS: fetal bovine serum
APC/C: anaphase-promoting complex/cyclosome
SAC: spindle assembly checkpoint
DAPI: 4',6-diamidino-2-phenylindole

## Supplementary Material

Supplementary Informations

## References

[bib1] Song Y, Brady ST. Post-translational modifications of tubulin: pathways to functional diversity of microtubules. Trends Cell Biol 2015; 25: 125–136. 25468068 10.1016/j.tcb.2014.10.004 PMC4344850

[bib2] Kull FJ, Sloboda RD. A slow dance for microtubule acetylation. Cell 2014; 157: 1255–1256. 24906144 10.1016/j.cell.2014.05.021

[bib3] Kavallaris M. Microtubules and resistance to tubulin-binding agents. Nat Rev Cancer 2010; 10: 194–204. 20147901 10.1038/nrc2803

[bib4] Ledizet M, Piperno G. Cytoplasmic microtubules containing acetylated alpha-tubulin in *Chlamydomonas reinhardtii*: spatial arrangement and properties. J Cell Biol 1986; 103: 13–22. 3722261 10.1083/jcb.103.1.13 PMC2113809

[bib5] Kline-Smith SL, Walczak CE. Mitotic spindle assembly and chromosome segregation: refocusing on microtubule dynamics. Mol Cell 2004; 15: 317–327. 15304213 10.1016/j.molcel.2004.07.012

[bib6] Holland AJ, Cleveland DW. Boveri revisited: chromosomal instability, aneuploidy and tumorigenesis. Nat Rev Mol Cell Biol 2009; 10: 478–487. 19546858 10.1038/nrm2718 PMC3154738

[bib7] Kops GJPL, Weaver BAA, Cleveland DW. On the road to cancer: Aneuploidy and the mitotic checkpoint. Nat Rev Cancer 2005; 5: 773–785. 16195750 10.1038/nrc1714

[bib8] Dryden SC, Nahhas FA, Nowak JE, Goustin AS, Tainsky MA. Role for human SIRT2 NAD-dependent deacetylase activity in control of mitotic exit in the cell cycle. Mol Cell Biol 2003; 23: 3173–3185. 12697818 10.1128/MCB.23.9.3173-3185.2003 PMC153197

[bib9] Inoue T, Hiratsuka M, Osaki M, Yamada H, Kishimoto I, Yamaguchi S et al. SIRT2, a tubulin deacetylase, acts to block the entry to chromosome condensation in response to mitotic stress. Oncogene 2007; 26: 945–957. 16909107 10.1038/sj.onc.1209857

[bib10] Brown AL, Kay GF. Bex1, a gene with increased expression in parthenogenetic embryos, is a member of a novel gene family on the mouse X chromosome. Hum Mol Genet 1999; 8: 611–619. 10072429 10.1093/hmg/8.4.611

[bib11] Han C, Liu H, Liu J, Yin K, Xie Y, Shen X et al. Human Bex2 interacts with LMO2 and regulates the transcriptional activity of a novel DNA-binding complex. Nucleic Acids Res 2005; 33: 6555–6565. 16314316 10.1093/nar/gki964 PMC1298925

[bib12] Naderi A, Liu J, Hughes-Davies L. BEX2 has a functional interplay with c-Jun/JNK and p65/RelA in breast cancer. Mol Cancer 2010; 9: 111. 20482821 10.1186/1476-4598-9-111 PMC2881879

[bib13] Zhou X, Meng Q, Xu X, Zhi T, Shi Q, Wang Y et al. Bex2 regulates cell proliferation and apoptosis in malignant glioma cells via the c-Jun NH2-terminal kinase pathway. Biochem Biophys Res Commun 2012; 427: 574–580. 23022184 10.1016/j.bbrc.2012.09.100

[bib14] Cromer A, Carles A, Millon R, Ganguli G, Chalmel F, Lemaire F et al. Identification of genes associated with tumorigenesis and metastatic potential of hypopharyngeal cancer by microarray analysis. Oncogene 2004; 23: 2484–2498. 14676830 10.1038/sj.onc.1207345

[bib15] Garber ME, Troyanskaya OG, Schluens K, Petersen S, Thaesler Z, Pacyna-Gengelbach M et al. Diversity of gene expression in adenocarcinoma of the lung. Proc Natl Acad Sci USA 2001; 98: 13784–13789. 11707590 10.1073/pnas.241500798 PMC61119

[bib16] Mas VR, Maluf DG, Archer KJ, Yanek K, Kong X, Kulik L et al. Genes involved in viral carcinogenesis and tumor initiation in hepatitis C virus-induced hepatocellular carcinoma. Mol Med 2009; 15: 85–94. 19098997 10.2119/molmed.2008.00110 PMC2605622

[bib17] Brito DA, Rieder CL. Mitotic checkpoint slippage in humans occurs via cyclin B destruction in the presence of an active checkpoint. Curr Biol 2006; 16: 1194–1200. 16782009 10.1016/j.cub.2006.04.043 PMC2749311

[bib18] Shin HJ, Baek KH, Jeon AH, Park MT, Lee SJ, Kang CM et al. Dual roles of human BubR1, a mitotic checkpoint kinase, in the monitoring of chromosomal instability. Cancer Cell 2003; 4: 483–497. 14706340 10.1016/s1535-6108(03)00302-7

[bib19] Weaver BA, Cleveland DW. Decoding the links between mitosis, cancer, and chemotherapy: the mitotic checkpoint, adaptation, and cell death. Cancer Cell 2005; 8: 7–12. 16023594 10.1016/j.ccr.2005.06.011

[bib20] Singh BN, Zhang G, Hwa YL, Li J, Dowdy SC, Jiang SW. Nonhistone protein acetylation as cancer therapy targets. Expert Rev Anticancer Ther 2010; 10: 935–954. 20553216 10.1586/era.10.62 PMC3273412

[bib21] Inoue T, Nakayama Y, Yamada H, Li YC, Yamaguchi S, Osaki M et al. SIRT2 downregulation confers resistance to microtubule inhibitors by prolonging chronic mitotic arrest. Cell Cycle 2009; 8: 1279–1291. 19282667 10.4161/cc.8.8.8245

[bib22] Magiera MM, Janke C. Post-translational modifications of tubulin. Curr Biol 2014; 24: R351–R354. 24801181 10.1016/j.cub.2014.03.032

[bib23] Taschner M, Vetter M, Lorentzen E. Atomic resolution structure of human alpha-tubulin acetyltransferase bound to acetyl-CoA. Proc Natl Acad Sci USA 2012; 109: 19649–19654. 23071318 10.1073/pnas.1209343109 PMC3511736

[bib24] Kim GW, Li L, Gorbani M, You LY, Yang XJ. Mice lacking alpha-tubulin acetyltransferase 1 are viable but display alpha-tubulin acetylation deficiency and dentate gyrus distortion. J Biol Chem 2013; 288: 20334–20350. 23720746 10.1074/jbc.M113.464792 PMC3711300

[bib25] Liu X, Xiao W, Wang XD, Li YF, Han JH, Li YQ. The p38-interacting protein (p38IP) regulates G(2)/M progression by promoting alpha-tubulin acetylation via inhibiting ubiquitination-induced degradation of the acetyltransferase GCN5. J Biol Chem 2013; 288: 36648–36661. 24220028 10.1074/jbc.M113.486910 PMC3868776

[bib26] Howes SC, Alushin GM, Shida T, Nachury MV, Nogales E. Effects of tubulin acetylation and tubulin acetyltransferase binding on microtubule structure. Mol Biol Cell 2014; 25: 257–266. 24227885 10.1091/mbc.E13-07-0387 PMC3890346

[bib27] Janke C, Bulinski JC. Post-translational regulation of the microtubule cytoskeleton: mechanisms and functions. Nat Rev Mol Cell Biol 2011; 12: 773–786. 22086369 10.1038/nrm3227

[bib28] Maney T, Hunter AW, Wagenbach M, Wordeman L. Mitotic centromere-associated kinesin is important for anaphase chromosome segregation. J Cell Biol 1998; 142: 787–801. 9700166 10.1083/jcb.142.3.787 PMC2148171

[bib29] Peris L, Wagenbach M, Lafanechere L, Brocard J, Moore AT, Kozielski F et al. Motor-dependent microtubule disassembly driven by tubulin tyrosination. J Cell Biol 2009; 185: 1159–1166. 19564401 10.1083/jcb.200902142 PMC2712961

[bib30] Lacroix B, van Dijk J, Gold ND, Guizetti J, Aldrian-Herrada G, Rogowski K et al. Tubulin polyglutamylation stimulates spastin-mediated microtubule severing. J Cell Biol 2010; 189: 945–954. 20530212 10.1083/jcb.201001024 PMC2886356

[bib31] McNally K, Audhya A, Oegema K, McNally FJ. Katanin controls mitotic and meiotic spindle length. J Cell Biol 2006; 175: 881–891. 17178907 10.1083/jcb.200608117 PMC2064698

[bib32] Deakin NO, Turner CE. Paxillin inhibits HDAC6 to regulate microtubule acetylation, golgi structure, and polarized migration. J Cell Biol 2014; 206: 395–413. 25070956 10.1083/jcb.201403039 PMC4121979

[bib33] North BJ, Marshall BL, Borra MT, Denu JM, Verdin E. The human Sir2 ortholog, SIRT2, is an NAD+-dependent tubulin deacetylase. Mol Cell 2003; 11: 437–444. 12620231 10.1016/s1097-2765(03)00038-8

[bib34] Hubbert C, Guardiola A, Shao R, Kawaguchi Y, Ito A, Nixon A et al. HDAC6 is a microtubule-associated deacetylase. Nature 2002; 417: 455–458. 12024216 10.1038/417455a

[bib35] Nagai T, Ikeda M, Chiba S, Kanno S, Mizuno K. Furry promotes acetylation of microtubules in the mitotic spindle by inhibition of SIRT2 tubulin deacetylase. J Cell Sci 2013; 126: 4369–4380. 23886946 10.1242/jcs.127209

[bib36] Reed NA, Cai D, Blasius TL, Jih GT, Meyhofer E, Gaertig J et al. Microtubule acetylation promotes kinesin-1 binding and transport. Curr Biol 2006; 16: 2166–2172. 17084703 10.1016/j.cub.2006.09.014

[bib37] Gardner MK, Zanic M, Howard J. Microtubule catastrophe and rescue. Curr Opin Cell Biol 2013; 25: 14–22. 23092753 10.1016/j.ceb.2012.09.006 PMC3556214

[bib38] Palazzo A, Ackerman B, Gundersen GG. Cell biology: tubulin acetylation and cell motility. Nature 2003; 421: 230. 12529632 10.1038/421230a

[bib39] Suematsu T, Li Y, Kojima H, Nakajima K, Oshimura M, Inoue T. Deacetylation of the mitotic checkpoint protein BubR1 at lysine 250 by SIRT2 and subsequent effects on BubR1 degradation during the prometaphase/anaphase transition. Biochem Biophys Res Commun 2014; 453: 588–594. 25285631 10.1016/j.bbrc.2014.09.128

[bib40] Huang J, Huen MS, Kim H, Leung CC, Glover JN, Yu X et al. RAD18 transmits DNA damage signalling to elicit homologous recombination repair. Nat Cell Biol 2009; 11: 592–603. 19396164 10.1038/ncb1865 PMC2743127

[bib41] Kim HS, Kim SH, Park HY, Lee J, Yoon JH, Choi S et al. Functional interplay between Aurora B kinase and Ssu72 phosphatase regulates sister chromatid cohesion. Nat Commun 2013; 4: 2631. 24149858 10.1038/ncomms3631

