# Supplementary material for: Oncogenic microtubule hyperacetylation through BEX4-mediated sirtuin 2 inhibition
Source: Cell Death Dis. 2016 Aug 11;7(8):e2336–. doi: 10.1038/cddis.2016.240 (PMC5108325; doi:10.1038/cddis.2016.240)
Supplement: Supplementary Informations [file cddis2016240x1.docx]

**Supplementary Information**

**Supplementary Materials and Methods**

**Plasmid construction.** Full-length BEX4 was generated by PCR. Fragments encoding BEX4 were subcloned into pEGFP-C1 (Clontech, Seoul, Rep. of Korea) to generate GFP-fused BEX4 expression vector (pEGFP-BEX4). Doxycycline-inducible GFP-BEX4 expression vector (pTRE2 hyg-GFP-BEX4) was generated by subcloning the PCR product, including GFP-BEX4 fragments, into pTRE2 hyg vector (Clontech). Full-length human BEX4 was also subcloned into pGEX-KG (GST-BEX4) and pTAP (TAP-BEX4) for GST pull-down assay and tandem affinity purification, respectively. The pTAP plasmid was a generous gift from Hongtae Kim (Sungkyunkwan University, Suwon, South Korea). To specifically knockdown BEX4, BUB1B, MAD2L1 and SIRT2, shRNAs were cloned into pSuper puro (Oligoengine) containing H1 promoter, according to the manufacturer’s instructions. Oligonucleotides encoding a shRNA against BEX4 (5’-GGCCATACCTAATAGGCATAT-3’, 5’-GCACTATATGCGCTTCCAAAC-3’, and 5’-GCATATTGAGCACAATGAAG-3’), BUB1B (5'-GGAGATCCTCTACAAAGGG-3') [^1^](#_ENREF_1), MAD2L1 (5’-GGAAGAGUCGGGACCACAG-3’) [^2^](#_ENREF_2), SIRT2 (3’-CCTGTGGCTAAGTAAACCATA-3’) [^3^](#_ENREF_3) or the luciferase (5’-CATA CGCGGAATACTTCGA-3’) control were synthesized and the efficiencies of each shRNA were determined by immunoblotting. GFP-SIRT2 (WT) and GFP-SIRT2 (H187Y) expressing plasmid were kindly provided by Kensaku Mizuno (Tohoku University, Aoba-ku, Sendai, Japan). SIRT2 (WT) and SIRT2 (H187Y) were subcloned using EcoRI and NotI into pGEX-KG plasmid to generate GST-SIRT2 (WT) and GST-SIRT2 (H187Y) expressing plasmid, respectively.

**Transwell migration and invasion assays.** Migration assays were performed using uncoated cell culture inserts with 8-μm pores (Greiner Bio-One). HeLa cells expressing GFP or GFP-BEX4 were cultured for 24 h, harvested, and resuspended in serum-free medium at a density of 10^6^ cells/ml. The bottom chambers were filled with 750 μl of complete medium and the top chambers were filled with 100 μl of cell suspension. After 16 h of incubation, cells migrating to the lower surface of the filter were fixed with 100% methanol and stained with 0.05% crystal violet. Non-migratory cells on the top surface of the filter were removed, and the migrated cells were quantified spectrophotometrically at 490 nm. Invasion assays were carried out using cell invasion assay kits (Merck Millipore) according to the manufacturer’s instructions. The assay was performed in an invasion chamber consisting of a 24-well cell culture plate with 12-cell culture inserts. HeLa cells expressing GFP or GFP-BEX4 were cultured for 24 h, harvested, and suspended in serum-free medium added to the inserts. Each insert was placed in the lower chamber containing complete medium. After 24 h incubation, invasiveness was evaluated by staining the cells that had migrated through the extracellular matrix layer and adhered to the polycarbonate membrane at the bottom of the insert. The invading cells were microscopically observed and photographed.

**Immunoblot analysis, immunoprecipitation and immunofluorescence.** For immunoblot analysis, cells were lysed in a NE buffer or RIPA buffer. Equal amounts of protein were resolved by SDS-PAGE, transferred to nitrocellulose filters, blocked, and analyzed with antibodies. To differentiate polymerized and non-polymerized TUB, cells were lysed in MTB buffer and centrifuged at 12,000 **×** g for 20 min at 25°C. Supernatants were collected and remaining cell pellets were resuspended in MTB buffer, sonicated on ice for 10 s, and centrifuged at 12,000 × g for 20 min at 4°C. For immunoprecipitation from total cellular extracts, cells were resuspended in a TNN buffer. The lysates were centrifuged and supernatants were incubated with proper antibodies and normal IgG (control) overnight at 4°C, and then with protein A/G agarose beads (Incospharm, Daejeon, Rep. of Korea). For immunofluorescence studies, cells were fixed, permeabilized, and incubated with the appropriate primary/secondary antibody combination (Alexafluor 488 and 568; Invitrogen). Cells were stained with 4',6-diamidino-2-phenylindole (DAPI) to visualize DNA, and viewed under a 510 Meta confocal microscope (Carl Zeiss, Seoul, Rep. of Korea).

**Live-cell imaging.** Cells were transfected with H2B-RFP vector and cultured in 6 well plates. Live-cell imaging was performed using a Zeiss Axiovert 200M microscope equipped with a Tempcontrol 37-2 digital and a CTI-Controller 3700 digital (PECON, Erbach, Germany) set at 37°C and 5% CO_2_. Time-lapse phase contrast images and red fluorescence protein (RFP) signals were recorded in parallel at multiple stage positions every 3 min for 48 h. Images were exported as JPEG files using ZEN 2012 software (Carl Zeiss).

**Buffer recipe**

| Number | Name | Recipe |
| --- | --- | --- |
| 1 | NE buffer | Tris-HCl (50 mmol/l, pH 7.5), NaCl (150 mmol/l), NP-40 (1%), phenylmethylsulfonyl fluoride (PMSF; 1 mmol/l), and dithiothreitol (DTT; 1 mmol/l), and a protease inhibitor cocktail |
| 2 | RIPA buffer | Tris-HCl (50 mmol/l, pH 8.0), NaCl (150 mmol/l), SDS (0.1%), EDTA (1 mmol/l), NP-40 (1%), Triton X-100 (1%), PMSF (1 mmol/l), and DTT (1 mmol/l), and a protease inhibitor cocktail |
| 3 | MTB buffer | Tris-HCl (20 mmol/l, pH 6.8), NaCl (140 mmol/l), MgCl_2_ (1 mmol/l), EGTA (2 mmol/l), NP-40 (0.5%), PMSF (1 mmol/l), DTT (1 mmol/l), and paclitaxel (4 μmol/l), and a protease inhibitor cocktail |
| 4 | TNN buffer | Tris–HCl (50 mmol/l, pH 7.5), NaCl (150 mmol/l), NP-40 (1%), PMSF (1 mmol/l), DTT (1 mmol/l), and a protease inhibitor cocktail |
| 5 | STE buffer | Tris-HCl (10 mmol/l, pH 8.0), NaCl (150 mmol/l), Triton X-100 (2 %), EDTA (1 mmol/l), phenylmethyl sulfonyl fluoride (PMSF; 1 mmol/l), dithiothreitol (DTT; 2 mmol/l), and lysozyme (100 μg/ml), and a protease inhibitor cocktail |
| 6 | Hypotonic buffer | Tris-HCl (50 mmol/l, pH 7.4), and KCl (55 mmol/l) |
| 7 | Carnoy’s solution | Methanol (75%) and acetic acid (25%) |

**Supplementary References**

1 Shin HJ, Baek KH, Jeon AH, Park MT, Lee SJ, Kang CM *et al*. Dual roles of human BubR1, a mitotic checkpoint kinase, in the monitoring of chromosomal instability. *Cancer Cell* 2003; **4**: 483-497.

2 Jeong SJ, Shin HJ, Kim SJ, Ha GH, Cho BI, Baek KH *et al*. Transcriptional abnormality of the hsMAD2 mitotic checkpoint gene is a potential link to hepatocellular carcinogenesis. *Cancer Res* 2004; **64**: 8666-8673.

3 North BJ, Rosenberg MA, Jeganathan KB, Hafner AV, Michan S, Dai J *et al*. SIRT2 induces the checkpoint kinase BubR1 to increase lifespan. *EMBO J* 2014; **33**: 1438-1453.

**Supplementary Figure Legends**

**Supplementary Figure S1** Generation of antibodies against BEX4. (**a**) To test the specificity of generated anti-BEX4 antibodies, 293T cell lines were transfected with the empty vector (Myc), Myc-tagged human BEX4 (Myc-hBEX4), or Myc-tagged mouse BEX4 (Myc-mBEX4) expressing vectors. Cell extracts were resolved by SDS-PAGE and immunoblotted with generated rabbit polyclonal anti-BEX4 antibody. (**b**) U251 cells were transfected with shLuc or two different set of shBEX4, shBEX4 #192, and shBEX4 #300, and cell extracts were immunoblotted using mouse polyclonal anti-BEX4 antibody in the presence or absence of BEX4 peptide. (**c**) U2OS cells were transfected with shLuc, shBEX4 #192, or shBEX4 #300 and cell extracts were immunoblotted using mouse polyclonal anti-BEX4 antibody. (**d**) 293T cells were transfected with expression plasmids encoding shLuc, shBEX4 #192, or shBEX4 #300. Transfected cells were harvested, lysed, and immunoblotted. Increasing amounts of protein extract from shLuc transfected cells were loaded for protein quantification (100%, 50%, and 25%, respectively). (**e**) HeLa cells were transfected with shLuc, shBEX4 #192, shBEX4 #300, or both shBEX4 #192 and #300 in combination with Myc-tagged BEX4 expression plasmid. Cells were lysed and immunoblotted with anti-c-Myc antibody.

**Supplementary Figure S2** Subcellular localization of BEX4 during the cell cycle. (**a**) HeLa cells were cultured, fixed with methanol, and co-stained with anti-α-TUB, and anti-BEX4. DNA was visualized by DAPI staining. Scale bars represent 5 μm. (**b**) HeLa cells were transfected with shLuc or shBEX4, and cells were fixed and co-stained with anti-α-TUB, and anti-BEX4. DNA was visualized by DAPI staining. Scale bars represent 5 μm.

**Supplementary Figure S3** Immunohistochemical analysis for BEX4 in normal lung tissues. (**a-b**) Immunohistochemical (IHC) staining using anti-BEX4 in pneumocytes (a) and respiratory epithelial cells (b) from normal lung tissues served as controls. These two normal lung tissues were assessed as IHC score 3 and stained in cytoplasm.

**Supplementary Figure S4** SIRT2 inhibition with mitotic spindle damage leads to the marked accumulation of aneuploid cells similar to BEX4 overexpression. (**a**) HeLa cells were transfected with expression plasmids encoding GFP and GFP-BEX4 protein and cultured in presence of nocodazole (200 ng/ml). At 36 h post-transfection, cells were lysed and immunoblotted with anti-acetylated-α-TUB (Ac-α-TUB), anti-α-TUB, anti-SIRT2, phosphor-Histone H3 Serine 28 [phospho-H3(S28)], anti-GFP, and anti-ACTB. The numbers at the bottom of blots indicate the relative intensity of bands normalized versus ACTB expression. (**b**) HeLa cells were treated with nocodazole (100 ng/ml) with or without sirtinol and AGK2. At the times indicated, cells were harvested, and stained with propidium iodide. DNA contents were analyzed by flow cytometry. (**c**) Histograms summarizing the distribution of different DNA contents in cells analyzed in B. Results are the mean ± s.e.m of three independent experiments. (**d**) HeLa cells were treated with nocodazole (100 ng/ml) with or without sirtinol and AGK2 for 18 h. Cells were lysed and immunoblotted with anti-acetylated-α-TUB (Ac-α-TUB), anti-α-TUB, anti-SIRT2, and anti-ACTB. The numbers at the bottom of blots indicate the relative intensity of bands normalized versus ACTB expression.

**Supplementary Figure S5** BALB/3T3 cells for *in vivo* tumorigenesis assay. BALB/3T3 (immortalized mouse fibroblast cell line) cells were transduced with retrovirus expressing a control GFP or GFP plus HA-tagged BEX4. At 72 h post-transduction, quantification of GFP positive cells was analyzed by flow cytometry.
