# Supplementary material for: Oncogenic microtubule hyperacetylation through BEX4-mediated sirtuin 2 inhibition
Source: Cell Death Dis. 2016 Aug 11;7(8):e2336–. doi: 10.1038/cddis.2016.240 (PMC5108325; doi:10.1038/cddis.2016.240)
Supplement: Supplementary Figure 1 [file cddis2016240x2.ppt]

## Slide 1
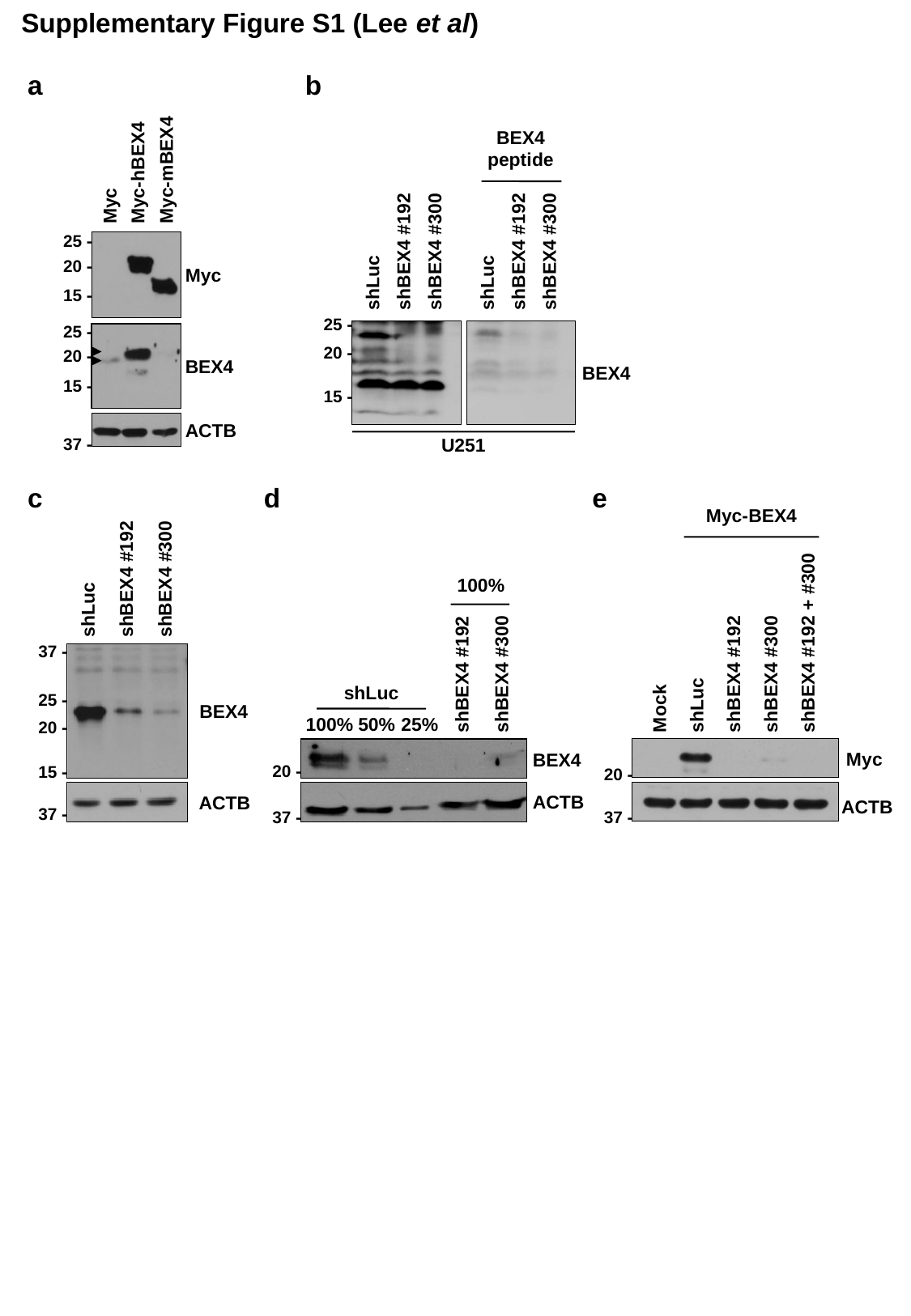

Supplementary Figure S1 (Lee et al)
a
b
BEX4
peptide
Myc-mBEX4
Myc-hBEX4
Myc
25 -
shBEX4 #300
shBEX4 #192
shBEX4 #300
shBEX4 #192
shLuc
shLuc
20 -
Myc
15 -
25 -
25 -
20 -
20 -
BEX4
BEX4
15 -
15 -
ACTB
37 -
U251
c
d
e
Myc-BEX4
shBEX4 #300
shBEX4 #192
shLuc
100%
shBEX4 #192 + #300
37 -
shBEX4 #192
shBEX4 #300
shBEX4 #300
shBEX4 #192
Mock
shLuc
shLuc
25 -
BEX4
100%
50%
25%
20 -
BEX4
Myc
20 -
15 -
20 -
ACTB
ACTB
ACTB
37 -
37 -
37 -
