# Supplementary material for: Oncogenic microtubule hyperacetylation through BEX4-mediated sirtuin 2 inhibition
Source: Cell Death Dis. 2016 Aug 11;7(8):e2336–. doi: 10.1038/cddis.2016.240 (PMC5108325; doi:10.1038/cddis.2016.240)
Supplement: Supplementary Figure 2 [file cddis2016240x3.ppt]

## Slide 1
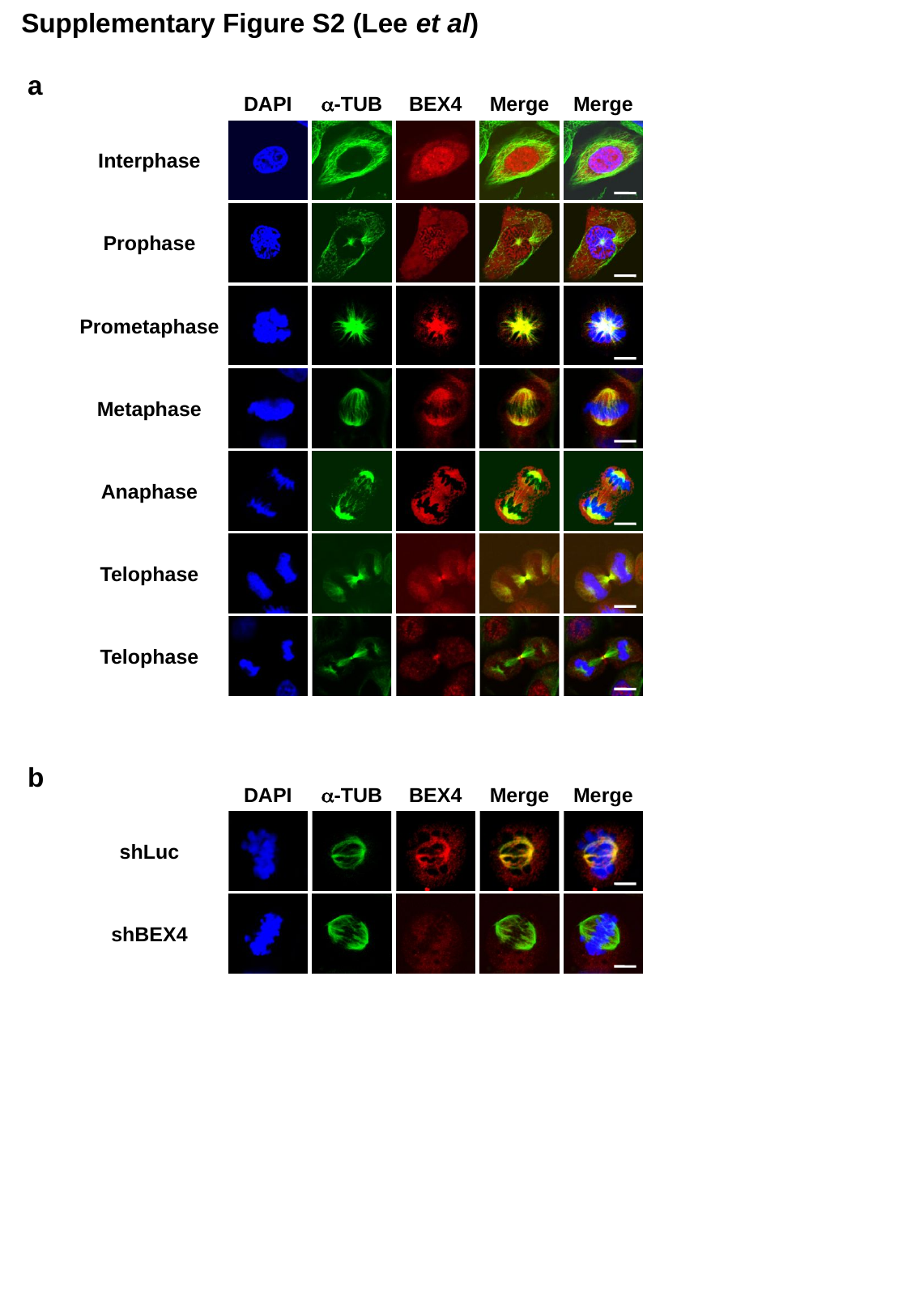

Supplementary Figure S2 (Lee et al)
a
DAPI
α-TUB
BEX4
Merge
Merge
Interphase
Prophase
Prometaphase
Metaphase
Anaphase
Telophase
Telophase
b
DAPI
α-TUB
BEX4
Merge
Merge
shLuc
shBEX4
