# Supplementary figures and images for: Oncogenic microtubule hyperacetylation through BEX4-mediated sirtuin 2 inhibition
Source: Cell Death Dis. 2016 Aug 11;7(8):e2336–. doi: 10.1038/cddis.2016.240 (PMC5108325; doi:10.1038/cddis.2016.240)

## Slide 1
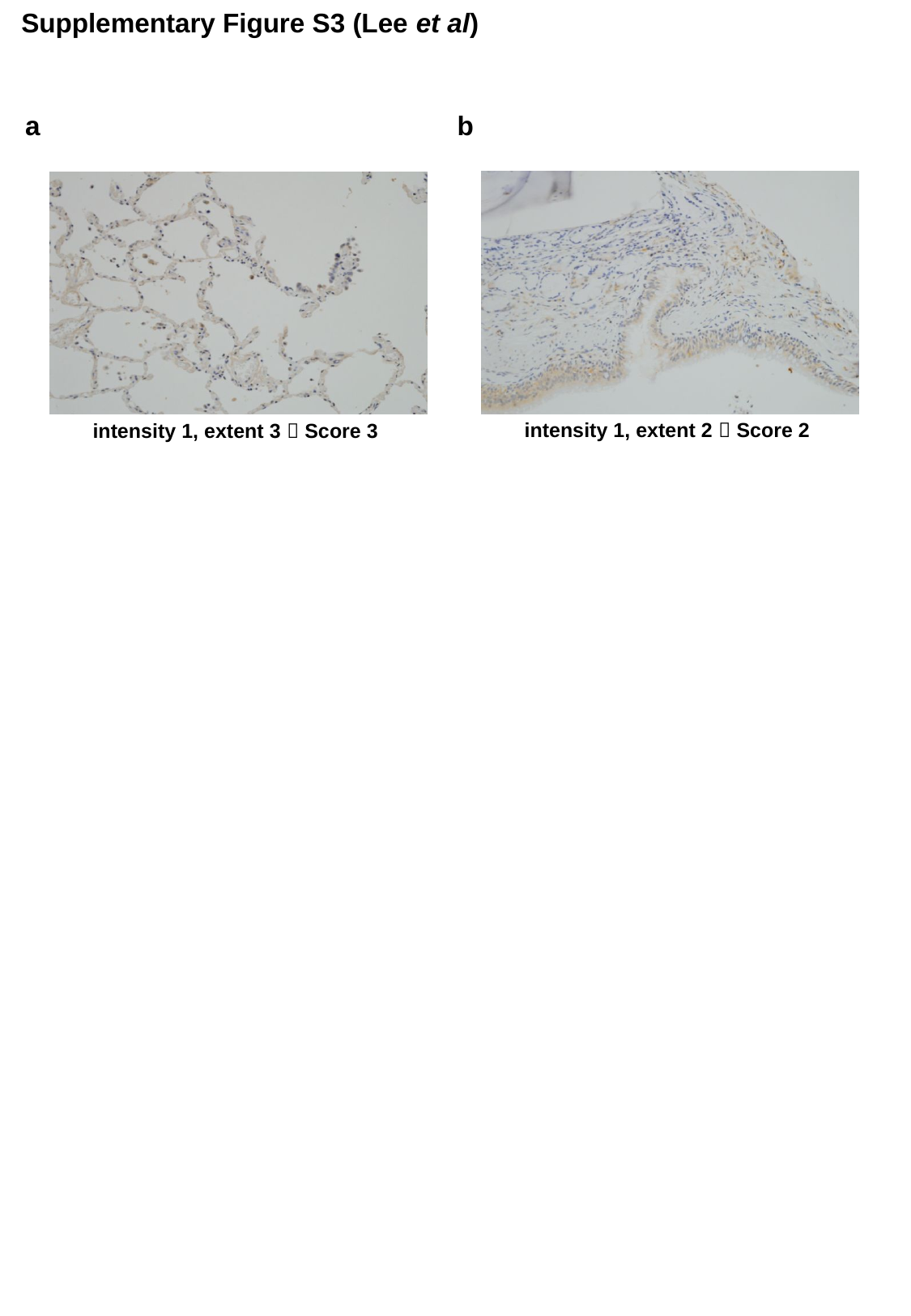

Supplementary Figure S3 (Lee et al)
a
b
intensity 1, extent 2  Score 2
intensity 1, extent 3  Score 3

Supplement: Supplementary Figure 3 [file cddis2016240x4.ppt]

## Slide 1
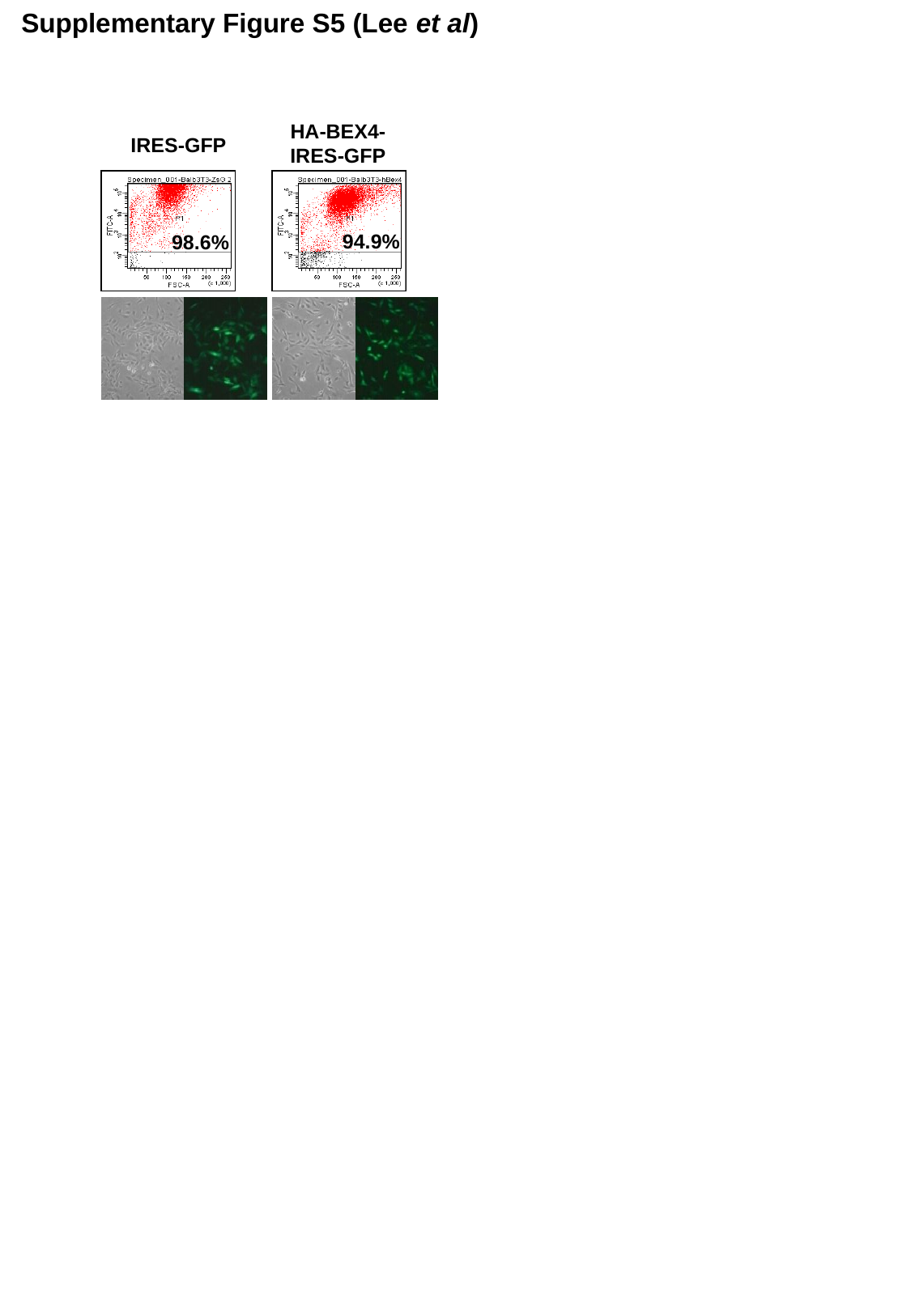

Supplementary Figure S5 (Lee et al)
HA-BEX4-
IRES-GFP
IRES-GFP
94.9%
98.6%

Supplement: Supplementary Figure 5 [file cddis2016240x6.ppt]
