# Supplementary material for: Oncogenic microtubule hyperacetylation through BEX4-mediated sirtuin 2 inhibition
Source: Cell Death Dis. 2016 Aug 11;7(8):e2336–. doi: 10.1038/cddis.2016.240 (PMC5108325; doi:10.1038/cddis.2016.240)
Supplement: Supplementary Figure 4 [file cddis2016240x5.ppt]

## Slide 1
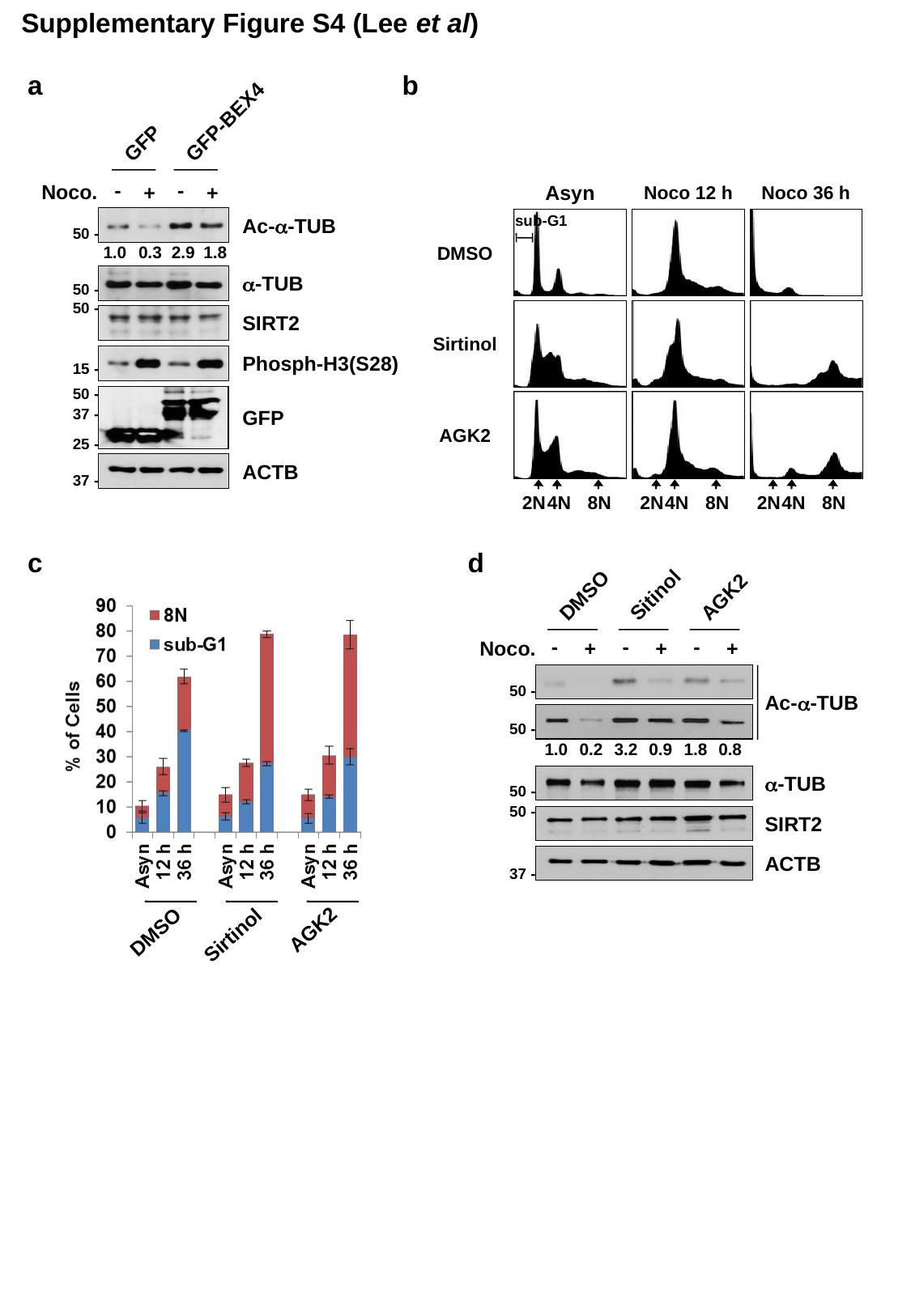

Supplementary Figure S4 (Lee et al)
a
b
GFP
GFP-BEX4
-
-
Noco.
+
+
Asyn
Noco 12 h
Noco 36 h
sub-G1
Ac-α-TUB
50 -
1.8
2.9
1.0
0.3
sub-G1
DMSO
α-TUB
50 -
50 -
SIRT2
Sirtinol
Phosph-H3(S28)
15 -
50 -
37 -
GFP
AGK2
25 -
ACTB
37 -
8N
8N
8N
2N
4N
2N
4N
2N
4N
c
d
Sitinol
DMSO
AGK2
-
-
-
Noco.
+
+
+
50 -
Ac-α-TUB
50 -
1.0
0.2
3.2
0.9
1.8
0.8
α-TUB
50 -
50 -
SIRT2
ACTB
37 -
AGK2
DMSO
Sirtinol
